# Marginal differences play a central role in complex leaf development

**DOI:** 10.1093/plcell/koaa010

**Published:** 2020-11-20

**Authors:** Chris Whitewoods

**Affiliations:** Department of Cell and Developmental Biology, John Innes Centre, Norwich

Development involves a delicate patterning of cell division and differentiation in three dimensions. This is beautifully illustrated in leaves, where three axes are visible: proximodistal (from base to tip), mediolateral (from the midvein to the margin/edge of the blade), and adaxial–abaxial (from upper to lower surface of the blade). Years of work have identified genes that control patterning along these axes, but how their activity is organized in time and space to control leaf development is less well understood.

In this issue of *The Plant Cell*, Martinez et al. (2021) address this problem by investigating the genetic mechanisms that pattern cell division and differentiation across a developing leaf. The authors began by characterising the 4th youngest leaf emerging from the tomato apical meristem (P4 stage), where the mediolateral and proximal–distal axes have just formed and several developing leaflets are visible.

They compared the development of wild type (WT) leaves with those of the *trifoliate (tf-2)* mutant, which produces only three leaflets—far fewer than WT. This allowed the authors to disentangle differentiation patterns due to leaf age (shared between WT and mutant) from those due to morphogenetic potential (much reduced in *tf-2*). When they visualized cell divisions by staining developing leaves with 5-ethynyl-29-deoxy-uridine (EdU), they found that cell divisions are higher in the margin compared to the rachis, but *tf-2* mutants have fewer cell divisions where additional leaflets develop in WT, suggesting that *TF* promotes cell division during leaflet formation.

**Figure 1:**
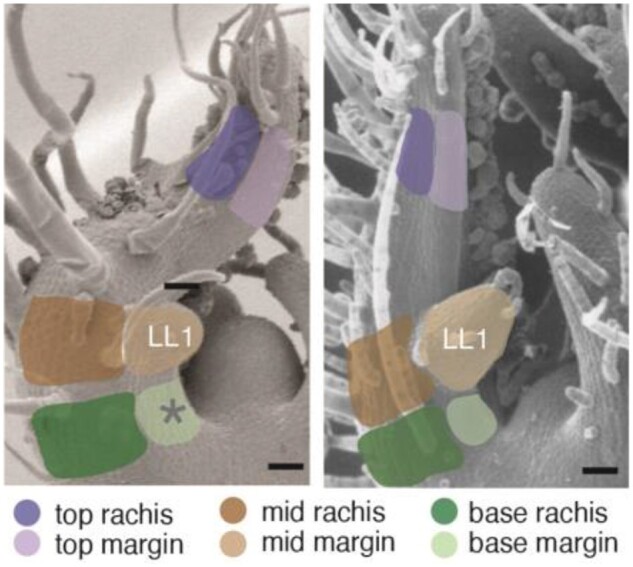
Leaf regions sampled for RNAseq. The authors sampled six regions along the proximodistal and mediolateral axes to identify factors controlling developmental patterning in leaves. Left panel, WT; right panel, *tf-2* mutant. (*Reprinted from*Martinez et al. [2021], *Figure 1.*)

They next used RNAseq to analyze transcriptional patterning across the proximodistal and mediolateral axes in WT. Using laser capture microdissection they sampled six regions of the P4 leaf: top, mid-region, and base along the proximodistal axis, each of which was divided along the mediolateral axis into rachis vs. margin (see Figure 1). This revealed complex patterns of genetic regulation, where genes involved in cellular growth processes are upregulated in the margin and sugar biosynthesis and transport are upregulated in the rachis. This mediolateral pattern is superimposed on a proximodistal pattern where photosynthetic genes are upregulated at the leaf tip, and morphogenetic genes at the leaf base. To confirm the RNAseq findings, the authors generated GUS reporter lines of the *Light Harvesting Chlorophyll A-B binding* (*CAB*) genes and confirmed that they are upregulated in the rachis as predicted.

To characterize detailed expression differences between the rachis and margin, the authors used self-organizing maps analysis to generate clusters of genes that are coregulated differentially between rachis and margin. In the margin, this revealed many genes known to be involved in margin identity, as well as genes related to auxin transport, biosynthesis, and regulation. They hypothesized that loss of auxin response in the margin may underlie the reduced ability of the *tf-2* mutant to make leaflets, and tested this by visualizing regions of high auxin response using the DR5:Venus reporter as well as localization of the *SlPIN1* auxin transporter. The expression of both was reduced and more diffuse in the *tf-2* mutant, suggesting the lack of leaflets may be due to reduced auxin response.

They compared expression differences between WT and *tf-2* and showed that the gene *Blade on Petiole* (*SlBOP2*) is significantly upregulated in the margins of *tf-2*. To test the functional role of *SlBOP2*, the authors analyzed *Slbop2* mutants and showed that leaves produce ectopic meristems and more leaflets, as previously demonstrated (Xu et al., 2016). Finally, they identified a *TF* binding site in the *SlBOP2* promoter, suggesting that *TF* may promote leaflet formation by regulating *SlBOP2* expression.

As well as painting a detailed picture of specific genetic regulators, these findings show that many genes expressed in the leaf margin are related to regulators of the shoot apical meristem. More work is needed to understand exactly how these genes have evolved to control diverse developmental processes, but this study leaves no doubt that the margin plays a central role in leaf morphogenesis.
